# Unique Urchin-like Ca_2_Ge_7_O_16_ Hierarchical Hollow Microspheres as Anode Material for the Lithium Ion Battery

**DOI:** 10.1038/srep11326

**Published:** 2015-06-10

**Authors:** Dan Li, Chuanqi Feng, Hua Kun Liu, Zaiping Guo

**Affiliations:** 1 Hubei Collaborative Innovation Center for Advanced Organic Chemical Materials, College of Chemistry and Chemical Engineering, Hubei University, Wuhan 430062, China; 2 Institute for Superconducting and Electronic Materials, School of Mechanical, Materials and Mechatronics Engineering, University of Wollongong, North Wollongong, NSW 2500, Australia

## Abstract

Germanium is an outstanding anode material in terms of electrochemical performance, especially rate capability, but its developments are hindered by its high price because it is rare in the crust of earth, and its huge volume variation during the lithium insertion and extraction. Introducing other cheaper elements into the germanium-based material is an efficient way to dilute the high price, but normally sacrifice its electrochemical performance. By the combination of nanostructure design and cheap element (calcium) introduction, urchin-like Ca_2_Ge_7_O_16_ hierarchical hollow microspheres have been successfully developed in order to reduce the price and maintain the good electrochemical properties of germanium-based material. The electrochemical test results in different electrolytes show that ethylene carbonate/dimethyl carbonate/diethyl carbonate (3/4/3 by volume) with 5 wt% fluoroethylene carbonate additive is the most suitable solvent for the electrolyte. From the electrochemical evaluation, the as-synthesized Ca_2_Ge_7_O_16_ hollow microspheres exhibit high reversible specific capacity of up to 804.6 mA h g^−1^ at a current density of 100 mA g^−1^ after 100 cycles and remarkable rate capability of 341.3 mA h g^−1^ at a current density of 4 A g^−1^. The growth mechanism is proposed based on our experimental results on the growth process.

Driven by the increasing need for flexible and portable flexible electronic devices, lithium ion batteries have attracted great efforts to develop advanced electrode materials with high electromotive force, energy density, and power density. To further increase the gravimetric and volumetric lithium storage capacity of lithium ion batteries, alloy-type anodes, especially group IVA elements, have been intensively explored as promising alternative active materials to conventional carbonaceous anode because of their high theoretical capacities^1^,^2^. For instance, metallic germanium has recently been widely considered as a promising anode material candidate because of its higher theoretical specific capacity of 1600 mA h g^−1^, and high lithium diffusivity and electrical conductivity^3^,^4^,^5^,^6^. On the other hand, germanium suffers from large volume changes of as much as 300% during full lithium insertion/extraction processes (corresponding to Li_4.4_Ge alloy)^7^,^8^, which results in structural collapse and deterioration of the cycling performance. To circumvent this problem, some structural or textural modifications are applied to improve the cycling performance of the germanium electrodes, such as nanosized structure^9^,^10^, highly porous structure^11^, and enclosing the germanium in a carbon matrix^12^,^13^. An alternative method is to synthesize binary or ternary germanium compounds^14^,^15^, which can form lithium compounds *in situ* during the initial lithium uptake process to serve as buffer matrices, although binary germanium compounds exhibit relatively low specific capacities^16^.

Ternary germanates, especially Ca_2_Ge_7_O_16_, can lower the expense for applications by reducing the germanium content compared to germanium electrode materials, and these ternary germanates have been recently investigated as anode materials for lithium ion batteries^6^,^16^. Importantly, the metal oxide formed *in situ* (CaO for Ca_2_Ge_7_O_16_) after the initial de-lithiation process, accompanied by the formation of Li_2_O, can not only serve as a buffer matrix to accommondate the volume changes in the germanium nanoparticles, but also effectively prevent the agglomeration of the nanosized germanium particles that are formed during the process^16^. Furthermore, the advantages of Ca_2_Ge_7_O_16_, in particular, such as its high theoretical capacity of 990 mA h g^−1^^17^, its cheap starting materials, and the environmentally benign nature of CaO formed *in situ* after lithium intercalation^16^, have made Ca_2_Ge_7_O_16_ the most popular compound among the metal germanates studied.

The recently reported metal germanate compounds are in simple structures, such as nanoparticles^18^, nanorods^19^, or nanowires^6^,^16^. These primary nanostructures, however, suffer from side reactions with electrolytes because of their high surface area, which results in safety concerns and poor calendar life^15^. To address this problem, synthesis of hierarchical structures with internal nanosized building blocks is an effective way to reduce the occurrence of side reactions^20^. Furthermore, the integral microscale architecture can effectively suppress the aggregation of the nanosized active materials, while the nanoscale building blocks can increase the kinetics of lithium ions^21^. Germanate compounds with hierarchical structure have never been reported.

Hollow nano-/micro-structured materials have drawn intensive interest owing to their unique structure-determined physical and chemical properties, which endow them with great potential for various applications^6^,^22^,^23^,^24^. Hollow structures are widely applied in the synthesis of electrode materials for lithium ion batteries due to their particular advantages, such as high surface area and short pathways for lithium ion diffusion and electron transport^25^,^26^. Their large surface area provides good contact between the electrode materials and electrolyte, as well as more storage sites for lithium ions. Especially important, the hollow interiors can provide extra room to accomodate the volume changes of the materials during the lithiation/de-lithiation processes and thus alleviate structrual strain. Therefore, it is a very attractive goal to develop a facile and environmentally benign method to obtain Ca_2_Ge_7_O_16_ in hierarchical hollow structures for applications in lithium ion batteries with excellent prospects for high electrochemical performance.

Herein, urchin-like hollow structured Ca_2_Ge_7_O_16_ microspheres constructed from nanorods as primary building blocks were successfully synthesized by a low cost and reliable solvothermal reaction. The compositions of the solvent and surfactants were found to have a great influence on the structure and morphology of the Ca_2_Ge_7_O_16_. Analysis of the electrochemical performance in different electrolytes shows that ethylene carbonate/dimethyl carbonate/diethyl carbonate (3/4/3 by volume) with 5 wt% fluoroethylene carbonate additive is the most suitable solvent for the electrolyte. The electrochemical performance of these urchin-like Ca_2_Ge_7_O_16_ hierarchical hollow microspheres was evaluated, and the electrode containing them showed high reversible specific capacity of up to 804.6 mA h g^−1^ at a current density of 100 mA g^−1^ after 100 cycles and remarkable rate capability of 341.3 mA h g^−1^ at a current density of 4 A g^−1^.

## Experimental Section

### Materials Synthesis

The Ca_2_Ge_7_O_16_ hollow microspheres were prepared by the solvothermal method. In a typical experiment, 0.1175 g Ca(CH_3_COO)_2_·H_2_O, 0.2746 g GeO_2_ (in a stoichiometric ratio of 2:7), and urea and/or hexadecyl trimethyl ammonium bromide (CTAB) were added into a mixture of ethanol and de-ionized (DI) water with different ratios and left to stir for 1 hour. Then, 30 mL of the resultant mixed solution was decanted into a Teflon-lined autoclave, and then, thermally treated at 180 °C for 24 h in an oven. After the solvothermal reaction, the obtained Ca_2_Ge_7_O_16_ white powder was decanted by centrifugation and washed 3 times with a large amount of ethanol and de-ionized water before drying at 70 °C in a vacuum oven for 12 h.

For the synthesis of Ca_2_Ge_7_O_16_ nanowires, 0.1175 g Ca(CH_3_COO)_2_·H_2_O and 0.2746 g GeO_2_ were dispersed into 30 mL deionized water. After stirring for 1 h, the suspension was decanted into a Teflon-lined stainless steel autoclave, followed by a heat treatment at 180 °C for 24 h in an oven. The white powder was decanted by centrifugation and washed 3 times with a large amount of ethanol and de-ionized water before drying at 70 °C in a vacuum oven for 12 h.

### Characterization

Powder X-ray diffraction (Bruker, D8-Advance XRD) was used to analyse the crystalline phases of the resultant materials, which was carried out using Cu Kα radiation (*λ* = 1.5406 Å) from 2*θ* = 15° to 70°. For observations of the morphologies and structures, the Ca_2_Ge_7_O_16_ samples were characterized using field-emission scanning electron microscopy (FESEM; JEOL, JEM-6700F, 5 kV) and transmission electron microscopy (TEM), which was performed on a JEOL JEM-2010 analytical electron microscope using 200 keV accelerating voltage. The N_2_ adsorption and desorption isotherms were obtained using a Quantachrome Instruments device (Autosorb AS-6B).

### Electrochemical Measurements

The Ca_2_Ge_7_O_16_ electrode slurry was prepared by thoroughly mixing the active material (80 wt%), carbon black (Super-P, 10 wt%), and poly(vinylidene fluoride) (PVDF) binder (10 wt%) in *N*-methyl pyrrolidone to prepare the working electrode. The resultant homogeneous slurry was then spread onto copper foil substrates. The electrodes were dried in a vacuum oven at 120 °C for 12 h prior to cell assembly. The cells were constructed of the prepared electrode as cathode, microporous polyethylene (Celgard 2400) as the separator, and lithium foil as anode. The electrolyte solutions investigated were 1.0 M LiPF_6_ in four different mixture of solvents, including ethylene carbonate/dimethyl carbonate/diethyl carbonate (EC/DMC/DEC, 1/1/1 by volume), EC/DMC/DEC (1/1/1 by volume) with 2 wt% vinylene carbonate (VC) additive, EC/DMC/DEC (3/4/3 by volume), EC/DMC/DEC (3/4/3 by volume) with 5 wt% fluoroethylene carbonate (FEC) additive. The whole assembly process for the CR2032 coin type cells was carried out in an argon-filled glove box. The discharge/charge cycling was performed within the voltage window between 0.02 and 3.0 V *vs.* Li^+^/Li using a battery test instrument (Land) at room temperature. The loading mass of active material was at least 1 mg cm^−2^ for all electrodes.

## Results and Discussion

In order to investigate the effects of the surfactants, the amounts of surfactants, and the composition of the solvothermal solution on the morphology of the obtained material, a series of experiments was designed and carried out. The surfactants are essential for the formation of hollow-structured microspheres. Without surfactants, the obtained germanate is composed of nanowires several tens to hundreds of micrometers in length with diameters in the range of 50–200 nm (See Figure S1 in the Supporting Information (SI)). The nanowire-structured morphology remained when hexadecyl trimethyl ammonium bromide (CTAB) was introduced into the synthesis system (as shown in Figure S2a). In contrast, the obtained samples are a mixture of microspheres and polyhedral particles when only urea is introduced (See Figure S2b). Hollow-structured Ca_2_Ge_7_O_16_ microspheres can be induced to form by the synergistic action of CTAB and urea. Furthermore, the ratio of ethanol to de-ionized water plays a significant role in the formation of well-defined Ca_2_Ge_7_O_16_ hollow microspheres. From the SEM images of materials synthesized with various ethanol/water ratios (See Figure S3), it can be found that when the ratio is up to 1:5, the hollow structure microspheres become more uniform, with a mean diameter of 2-3 μm. Increasing the ratio to 1:2 provides larger size microspheres. The spherical structure shifts to polyhedral with further increased ratios above 1:1.

**Figure 1:**
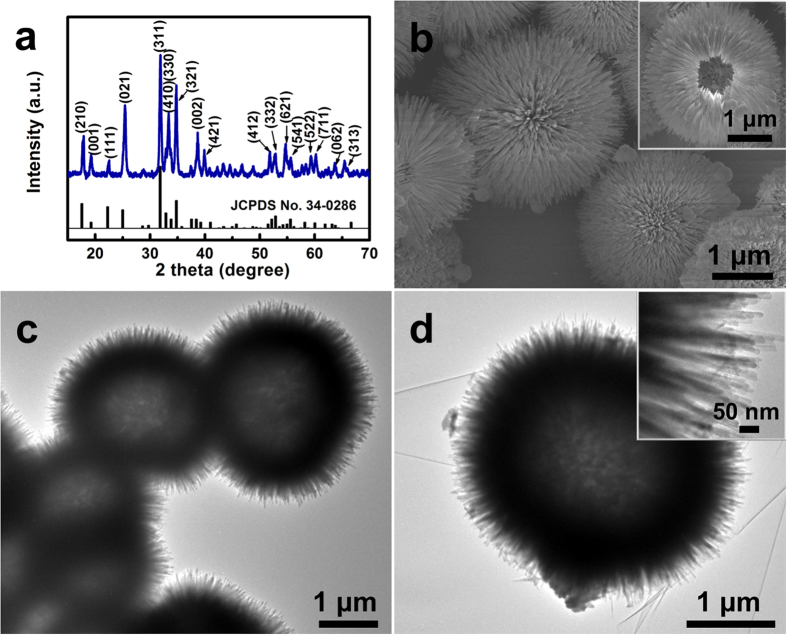
(**a**) Powder X-ray diffraction pattern, (**b**) SEM image, (**c**) and (**d**) TEM images of Ca_2_Ge_7_O_16_ hollow microspheres. The insets in panels (**b**) and (**d**) show the hollow void at the center of a Ca_2_Ge_7_O_16_ microsphere, and a TEM image of the magnified surface of a microspheres, respectively.

To understand the specific roles of the surfactants CTAB and urea, various amounts of the surfactants were also tested to observe the structural changes (Figures S4 and S5). The results obviously demonstrate that superfluous surfactants can ruin the hierarchical hollow structure. To sum up, uniform hollow Ca_2_Ge_7_O_16_ microspheres with numerous nanorods radially grown on the surface are obtained with 30 mmol urea and 4 mmol CTAB, with an ethanol/water ratio of 1:5. The crystal structure of the Ca_2_Ge_7_O_16_ hollow microspheres was characterized by X-ray diffraction (XRD), as shown in Fig. 1a. All the reflection peaks of the Ca_2_Ge_7_O_16_ hollow microspheres are well indexed to orthorhombic phase Ca_2_Ge_7_O_16_ (JCPDS card No. 34-0286). The hollow voids at the centres of the microspheres can be observed from the broken one in the SEM images (Fig. 1b). The hollow structure is further confirmed by the TEM image of the Ca_2_Ge_7_O_16_ microspheres shown in Fig. 1c,d. With a closer examination of a single sphere (as shown in the inset of Fig. 1d), the nanorod subunits, which are about 30 nm in width and about 300 nm in length, can be observed to have a uniform distribution around the circumference of the sphere, and a well-defined shell is formed, as indicated by the dark ring in the image. In the high-resolution transmission electron microscope (HRTEM) image shown in Figure S6, the crystal lattice fringes with *d*-spacing of 0.47 nm are characteristic of the (001) lattice planes of orthorhombic Ca_2_Ge_7_O_16_, indicating a preferred [001] growth direction^16^. As revealed by the nitrogen adsorption-desorption isotherms (Figure S7), the urchin-like microspheres with hollow structures feature a relatively high Brunauer-Emmett-Teller (BET) specific surface area of 64.7 m^2^ g^−1^ and a high pore volume of 0.48 cm^3^ g^−1^ with a broad pore-size distribution.

**Figure 2:**
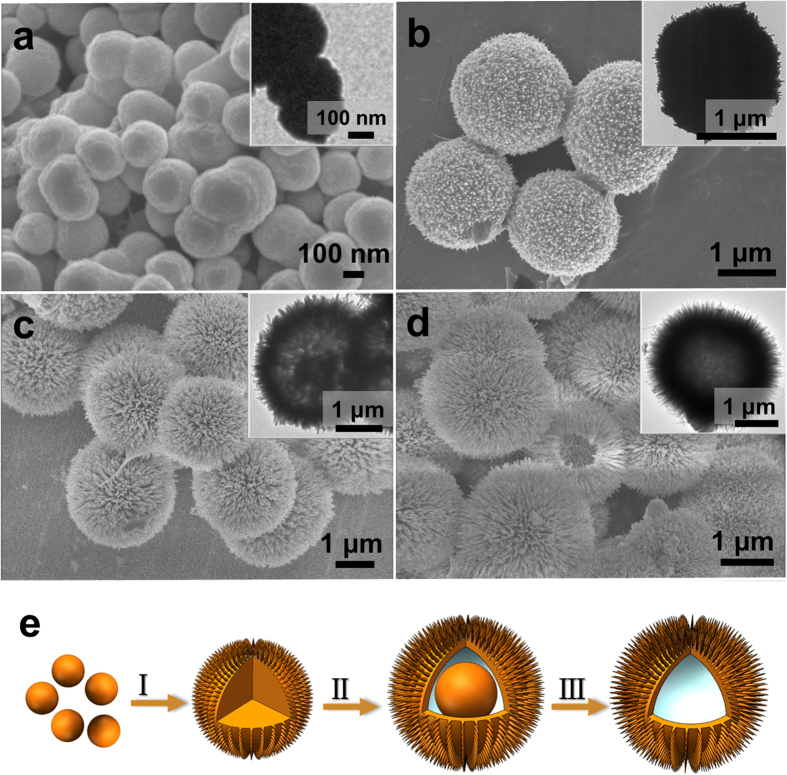
SEM and TEM images of the products obtained after reaction for (**a**) 30 min, (**b**) 50 min, (**c**) 10 h, and (**d**) 24 h, with the insets showing the corresponding bright field TEM images. (**e**) Schematic illustration of the morphological evolution process of the urchin-like Ca_2_Ge_7_O_16_ hollow microspheres: (I) self-assembly process, (II) inside-out Ostwald ripening and recrystallization process, and (III) thorough dissolution and recrystallization process.

A series of time-dependent experiments was carried out to fully understand the formation process and structural changes of the hollow microspheres. Figure 2 shows SEM and TEM images of samples subjected to different reaction times (30 min, 50 min, 10 h, and 24 h), indicating a fast formation process for the urchin-like microspheres. It can be observed that nanospheres were formed at the short reaction time of 30 min (shown in Fig. 2a). Just twenty minutes later, the spheres grew to microsize (about 1.5 μm), as shown in Fig. 2b, and uniform, but short and rather undeveloped tiny nanorods were assembled on the surfaces of the microspheres. Interestingly, yolk-shell structural microspheres (~2 μm in size) were obtained when the reaction time was extended to 10 h, as shown in Fig. 2c. This appearance of partial hollow voids and increased size distribution can be ascribed to the consumption of the interior core accompanied by recrystallization at the exterior surface of the microspheres and growth of the nanorods on the shells, according to the well-known inside-out Ostwald-ripening process^6^. When the reaction time was prolonged to 24 h, urchin-like microspheres with completed and well-defined hollow interiors were finally created, as shown in Fig. 2d. Based on the above morphology and structural change observations, the formation mechanism of the hierarchical urchin-like Ca_2_Ge_7_O_16_ hollow spheres is proposed (see Fig. 2e). Initially, OH^−^ ions released by the slow hydrolysis of calcium acetate and urea react with GeO_2_ to form soluble HGeO_3_^−^ anions. Then, small Ca_2_Ge_7_O_16_ nuclei are generated to form small nanosized spheres when the Ca^2+^ and HGeO_3_^−^ reach the supersaturation limit^6^,^16^. After a self-assembly process, the nanoparticles grow to solid microspheres with nanorods on the surfaces of the spheres in stage I. Then, the microspheres undergo inside-out Ostwald-ripening and recystallization processes, accompanied by further growth of the nanorods on the surfaces of the microspheres, leading to the formation of yolk-shell structures (as shown in stage II)^27^. Further lengthening of the reaction time results in thorough dissolution and recrystallization and completely hollow structured Ca_2_Ge_7_O_16_ microspheres with hierarchical urchin-like surfaces eventually formed (stage III).

**Figure 3:**
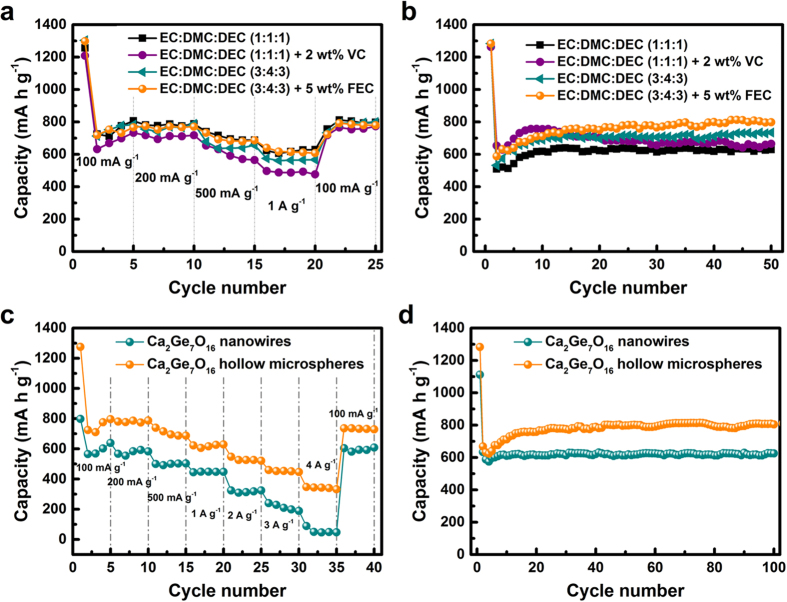
(**a**) Rate capability of Ca_2_Ge_7_O_16_ hollow microspheres in different electrolytes at different current densities. (**b**) Cycling performance of Ca_2_Ge_7_O_16_ hollow microspheres in different electrolytes under a current density of 100 mA g^−1^. (**c**) Comparison of rate capability of Ca_2_Ge_7_O_16_ nanowires and Ca_2_Ge_7_O_16_ hollow microspheres at different current densities in electrolyte with EC/DMC/DEC (3/4/3) + 5 wt% FEC. (**d**) Comparison of cycling performance of Ca_2_Ge_7_O_16_ nanowires and Ca_2_Ge_7_O_16_ hollow microspheres under a current density of 100 mA g^−1^ in electrolyte with EC/DMC/DEC (3/4/3) + 5 wt% FEC.

In order to fully understand the electrochemical behavior of the Ca_2_Ge_7_O_16_ hollow microsphere anode, the electrolyte for the testing cells was optimized. Figure 3a,b shows the influences of different electrolytes on the electrochemical performance of Ca_2_Ge_7_O_16_ hollow microsphere anode with 1 M LiPF_6_ in EC/DMC/DEC (1/1/1), EC/DMC/DEC (1/1/1) + 2 wt% VC, EC/DMC/DEC (3/4/3), and EC/DMC/DEC (3/4/3) + 5 wt% FEC. For the rate capability (shown in Fig. 3a), the cells with EC/DMC/DEC (1/1/1) and EC/DMC/DEC (3/4/3) + 5 wt% FEC show almost the same rate properties, which are better than those with EC/DMC/DEC (1/1/1) + 2 wt% VC and EC/DMC/DEC (3/4/3). There is no capacity drop in electrolytes consisting of EC/DMC/DEC (1/1/1) and EC/DMC/DEC (3/4/3) + 5 wt% FEC when the current density is increased from 100 to 200 mA g^−1^. The average capacity is 687.6 mA h g^−1^ at 500 mA g^−1^ and 628.6 mA h g^−1^ at 1 A g^−1^. Figure 3b compares the cycling performances of the cells in different electrolytes at a current density of 100 mA g^−1^. It can be observed that the capacity increases during cycling for the first 10 cycles for all the cells in different electrolytes, which can be attributed to electrochemical activation^16^. The cell with EC/DMC/DEC (1/1/1) + 2 wt% VC shows capacity fading after the 10^th^ cycle and drops to a capacity of 664.5 mA h g^−1^ at the 50^th^ cycle, while the cells in the other electrolytes remain relatively stable after the 10^th^ cycle. The cell with EC/DMC/DEC (3/4/3) + 5 wt% FEC exhibits the best cycling performance, with an increased capacity during cycling to 797.5 mA h g^−1^ at the 100^th^ cycle.

Using the optimized electrolyte, the electrochemical performance of Ca_2_Ge_7_O_16_ hollow microspheres anode was compared with that of Ca_2_Ge_7_O_16_ nanowires in electrolyte with EC/DMC/DEC (3/4/3) + 5 wt% FEC. Figure 3c presents the rate capabilities of Ca_2_Ge_7_O_16_ nanowires and Ca_2_Ge_7_O_16_ hollow microspheres at different current densities. The Ca_2_Ge_7_O_16_ hollow microspheres showed excellent rate capability. The average discharge capacities are 856.6 mA h g^−1^ under a current density of 100 mA g^−1^, 781.2 mA h g^−1^ under 200 mA g^−1^, 705.2 mA h g^−1^ under 500 mA g^−1^, 620.2 mA h g^−1^ under 1 A g^−1^, 529.3 mA h g^−1^ under 2 A g^−1^, and 452.6 mA h g^−1^ under 3 A g^−1^. Even at the high current density of 4 A g^−1^, the specific capacity remains 341.3 mA h g^−1^. When the discharge/charge current density was returned to 100 mA g^−1^, the specific capacity recovered to 732.8 mA h g^−1^, indicating the good reversibility of the Ca_2_Ge_7_O_16_ hollow microspheres. As for the Ca_2_Ge_7_O_16_ nanowires, the electrode presented inferior rate capability compared to the Ca_2_Ge_7_O_16_ hollow microspheres. Only a capacity of 56.1 mA h g^−1^ was delivered at a current density of 4 A g^−1^, with an extremely low retention of 9.7% of the average capacity at a current density of 200 mA g^−1^.

Figure 3d shows the cycling performances of the two Ca_2_Ge_7_O_16_ materials at a current density of 100 mA g^−1^ at room temperature within the voltage range of 0.02–3 V. The Ca_2_Ge_7_O_16_ hollow microspheres showed good cycling performance. The capacity fading in the first three cycles may be attributable to the irreversible reduction of Ca_2_Ge_7_O_16_ to germanium, accompanied by the formation of Li_2_O and CaO, as well as the formation of a solid electrolyte interphase (SEI). There is increasing capacity from the 4^th^ discharge cycle. The capacity is 804.6 mA h g^−1^ after 100 cycles, corresponding to 120.4% of the capacity at the second cycle, revealing superior reversible specific capacity and cycling performance. The capacity of the Ca_2_Ge_7_O_16_ nanowires, as shown in Fig. 3d, is stable with cycling, but much lower than that of the Ca_2_Ge_7_O_16_ hollow microspheres with increasing cycle number, with a value of 624.7 mA h g^−1^ at the 100^th^ cycle. The specific capacity, the cycling performance, and the rate capability of the as-prepared Ca_2_Ge_7_O_16_ hollow microspheres are better than those of previously reported Ca_2_Ge_7_O_16_ nanowires^16^. Figure S8 shows the galvanostatic charge–discharge profiles of the Ca_2_Ge_7_O_16_ hollow microspheres in the 1st, 2nd and 100th cycles under a current density of 100 mA g^−1^. The discharge distinct plateaus at 0.37 V and 0.15 V correspond to the formation of Li_7_Ge_2_and Li_22_Ge_5_ alloys related to the lithiation reaction, while the charge plateaus at 0.5 V correspond to the delithiation reaction^28^,^29^. The SEM images of Ca_2_Ge_7_O_16_ hollow microspheres electrode after 100 cycles are showed in Figure S9, indicating the good structural stability.

We believe that the superior electrochemical properties of our Ca_2_Ge_7_O_16_ hollow microspheres compared to those of Ca_2_Ge_7_O_16_ nanowires are attributable to the unique urchin-like hollow structures. Firstly, the hollow structured Ca_2_Ge_7_O_16_ micropheres were formed by a self-supported transformation process, which offers high structural integrity and stability, thus providing good cycling performance. Secondly, the hollow microspheres are composed of one-dimensional Ca_2_Ge_7_O_16_ nanorods as building blocks, which may facilitate the lithium ion diffusion along the longitudinal directions and shorten the diffusion paths for lithium ions. Thirdly, the hollow structure endows Ca_2_Ge_7_O_16_ with free space for the volume expansion of germanium nanoparticles in the process of lithium insertion and thus alleviates the strain stemming from the volume expansion. In the meanwhile, the hollow structures facilitate the penetration of the electrolyte and lithium ion transport in the electrode. Moreover, the high surface area of the Ca_2_Ge_7_O_16_ provides increased reactive sites and interfaces between the active materials and the electrolyte, leading to enhanced lithium storage capacity and high-rate capability.

## Conclusions

In summary, hierarchical urchin-like Ca_2_Ge_7_O_16_ hollow microspheres were synthesized by a solvothermal reaction in a system of ethanol-deionized water (1:5 by volume). The as-obtained hierarchical microspheres revealed a hollow interior and an urchin-like shell composed of numerous nanorods, which are about 30 nm in width and about 300 nm in length. The unique structure effectively alleviates the strain of volume change, provides a short lithium ion diffusion length, and facilitates the penetration of electrolyte, thereby maintaining the structural integrity and stability of the anode, and ensuring favorable transport kinetics for both lithium ions and electrons. Therefore, the urchin-like Ca_2_Ge_7_O_16_ hollow microspheres present high reversible capacity, and superior cycling stability and rate capability, exhibiting potential as a promising anode material for lithium ion batteries.

## Additional Information

**How to cite this article**: Li, D. *et al.* Unique Urchin-like Ca_2_Ge_7_O_16_ Hierarchical Hollow Microspheres as Anode Material for the Lithium Ion Battery. *Sci. Rep.* **5**, 11326; doi: 10.1038/srep11326 (2015).

## Supplementary Material

Supplementary Information
