# Supplementary material for: Unique Urchin-like Ca2Ge7O16 Hierarchical Hollow Microspheres as Anode Material for the Lithium Ion Battery
Source: Sci Rep. 2015 Jun 10;5:11326. doi: 10.1038/srep11326 (PMC4462153; doi:10.1038/srep11326)
Supplement: Supplementary Information [file srep11326-s1.pdf]

Supplementary Information

Unique Urchin-like $Ca_2Ge_7O_{16}$ Hierarchical Hollow Microspheres as Anode Material for the Lithium Ion Battery

Dan Li[a,b], Chuanqi Feng[a], Hua Kun Liu[b], Zaiping Guo [a,b,*]

[a]Hubei Collaborative Innovation Center for Advanced Organic Chemical Materials, College of Chemistry and Chemical Engineering, Hubei University, Wuhan 430062, China

[b]Institute for Superconducting and Electronic Materials, School of Mechanical, Materials and Mechatronics Engineering, University of Wollongong, North Wollongong, NSW 2500, Australia

E-mail: zguo@uow.edu.au; Tel: +61 2 4221 5225

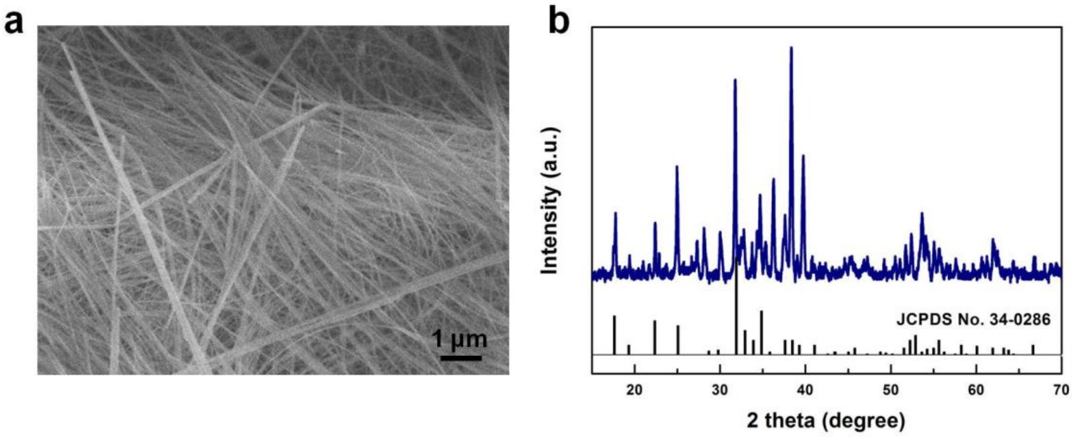

Figure S1. (a) SEM image and (b) powder X-ray diffraction pattern of as-prepared $Ca_2Ge_7O_{16}$ nanowires.

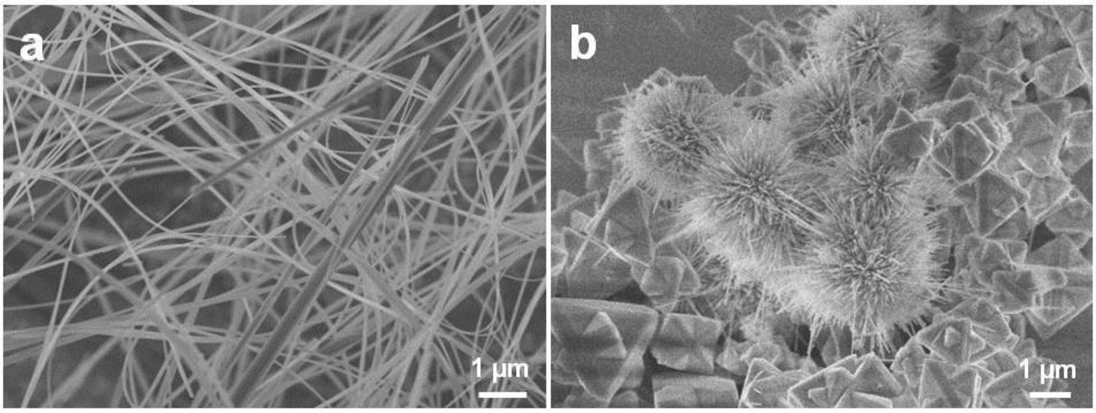

Figure S2. SEM image of as-prepared $Ca_2Ge_7O_{16}$ with (a) CTAB and (b) urea.

In a typical synthesis, 1.456 g CTAB (4 mmol) and 1.8 g urea (30 mmol) were introduced into the solvothermal reaction to obtain (a) sample A and (b) sample B, respectively.

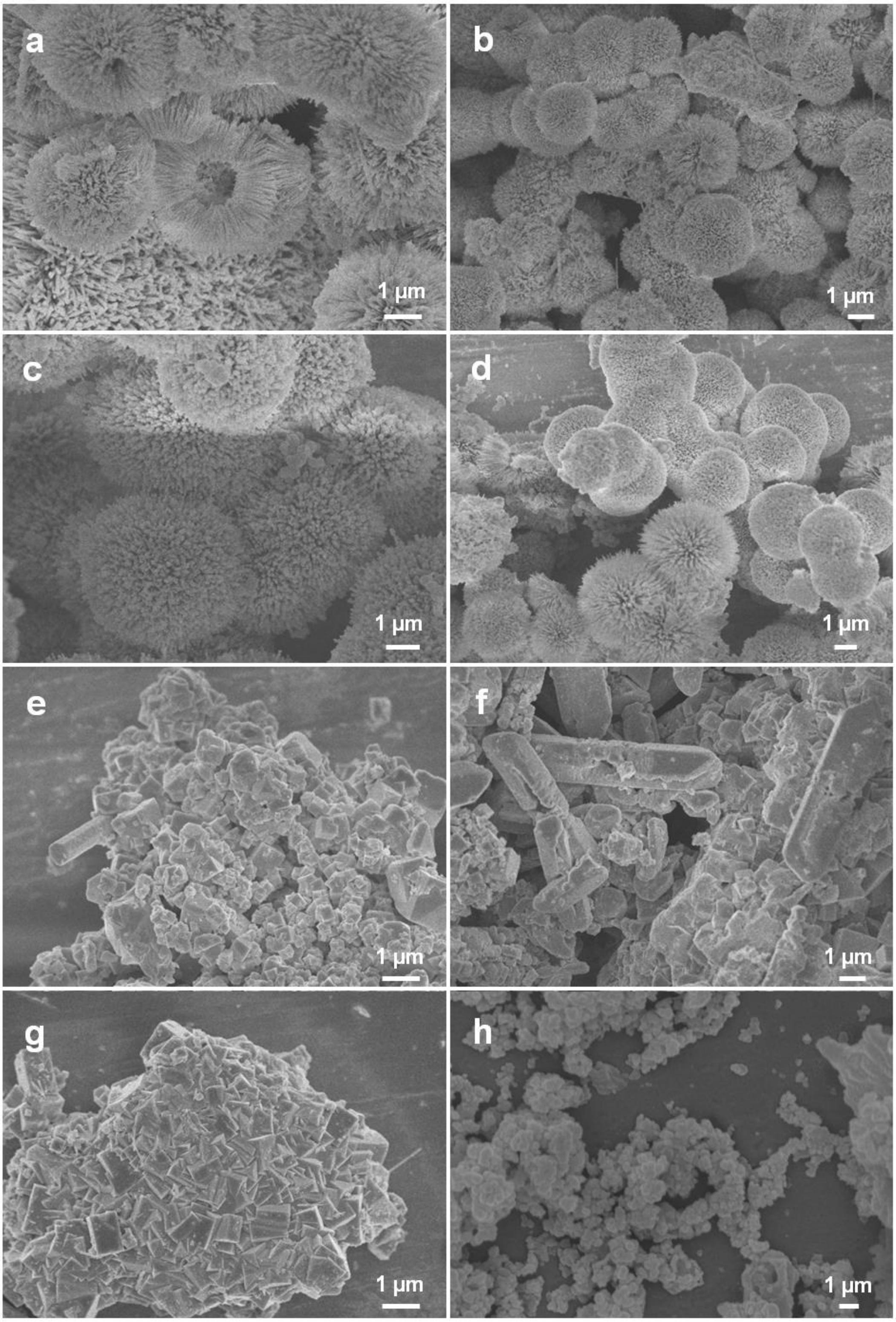

Figure S3. SEM images of $Ca_2Ge_7O_{16}$ materials prepared under different solvothermal conditions: (a) and (b) 5 mL ethanol and 25 mL de-ionized (DI) water; (c) and (d) 10 mL ethanol and 20 mL DI water; (e) 15 mL ethanol and 15 mL DI water; (f) 20 mL ethanol and 10 mL DI water; (g) 25 mL ethanol and 5 mL DI water; (h) 30 mL ethanol. with the other conditions unchanged.

The amount of ethanol was found to have an important influence on the formation of hollow structure and the morphology of the materials, which could be ascribed to its interactions with the calcium acetate and germanium dioxide. Furthermore, the ethanol/water ratio could affect the grow rate of the $Ca_2Ge_7O_{16}$ due to the different interfacial energies and solvent polarities, which leading to the various morphologies of the materials[1].

1. Kim, C. & Kwon, M. Effects of water content in an ethanol-based solvent on sol-gel synthesis of Zn_2SiO_4:Mn phosphors. *Electronic Materials Letters* **5**, 113-117 (2009).

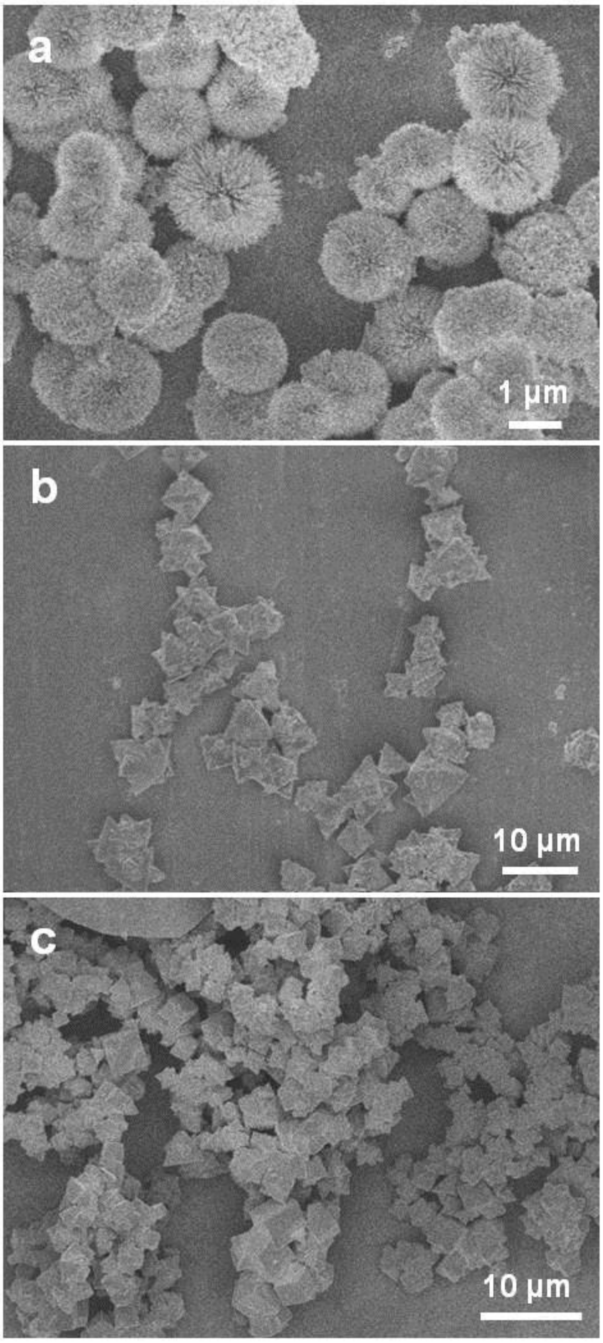

Figure S4. SEM images of $Ca_2Ge_7O_{16}$ materials prepared using different amounts of urea: (a) 1.8 g (30 mmol), (b) 3.6 g (60 mmol), (c) 5.4 g (90 mmol), all in a mixed solution of 5 mL ethanol and 25 mL DI water.

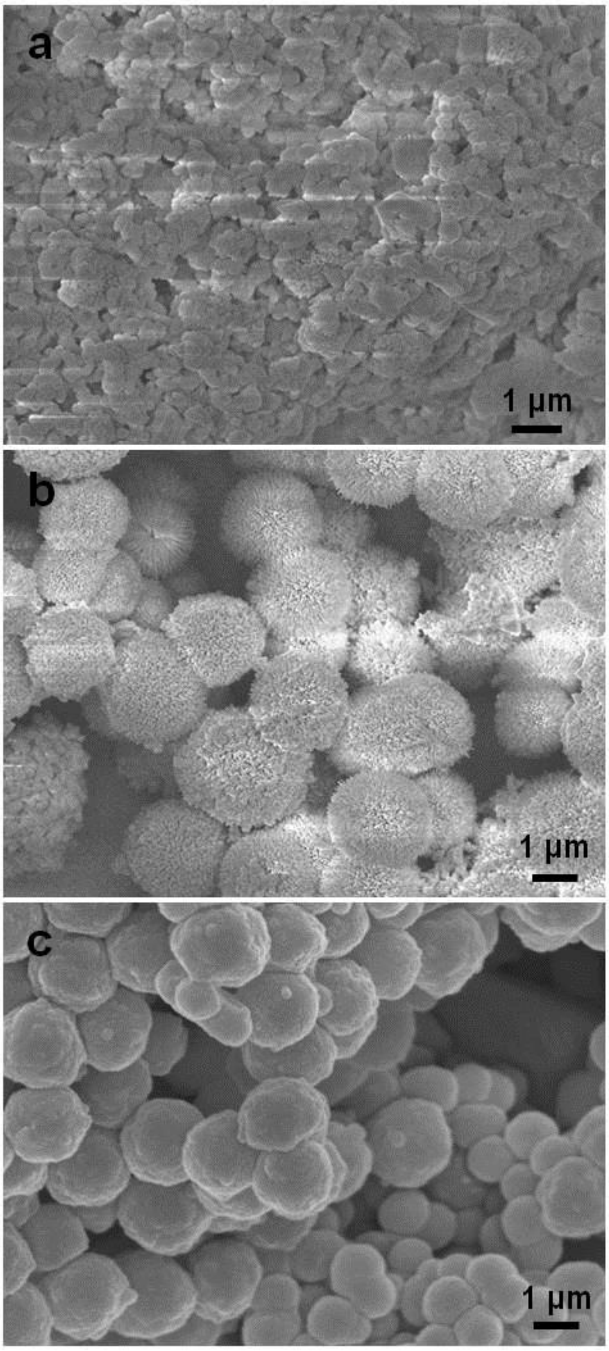

Figure S5. SEM images of $Ca_2Ge_7O_{16}$ prepared using different amounts of CTAB: (a) 0.728 g (2 mmol), (b) 1.456 (4 mmol), (c) 2.184 g (6 mmol), all in a mixed solution of 5 mL ethanol and 25 mL DI water.

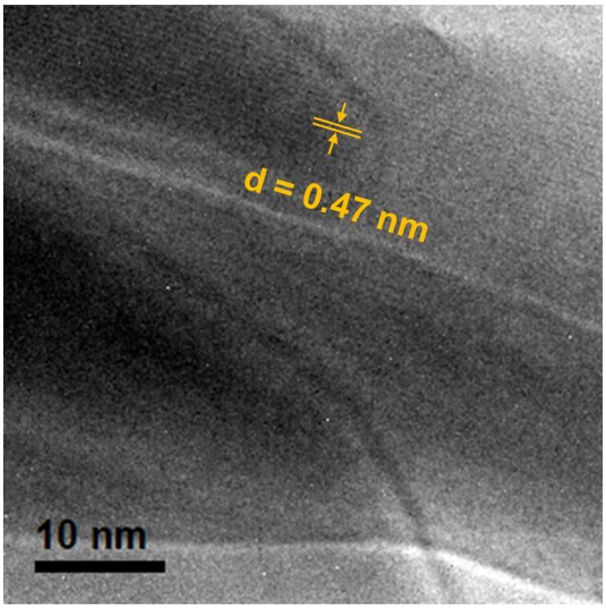

Figure S6. High-resolution TEM image of $Ca_2Ge_7O_{16}$ hollow microsphere.

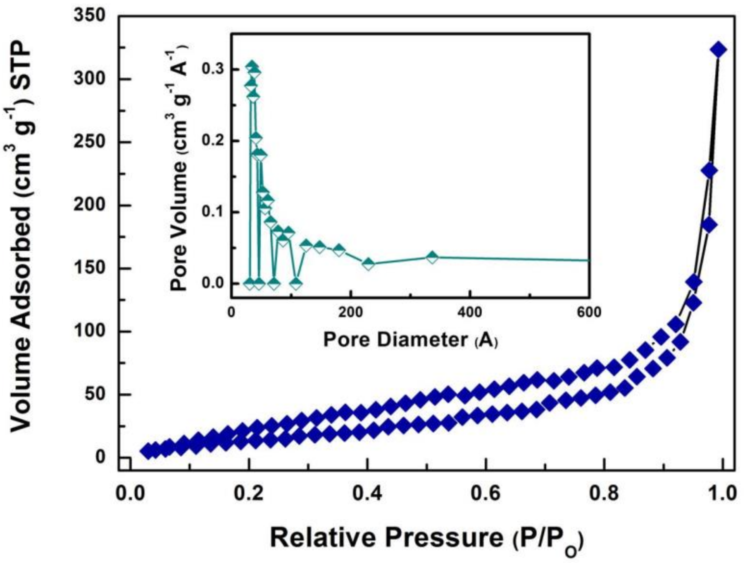

Figure S7. N_2 adsorption-desorption isotherm of $Ca_2Ge_7O_{16}$ hollow microspheres. The inset indicates the pore-size distribution from the corresponding desorption branch.

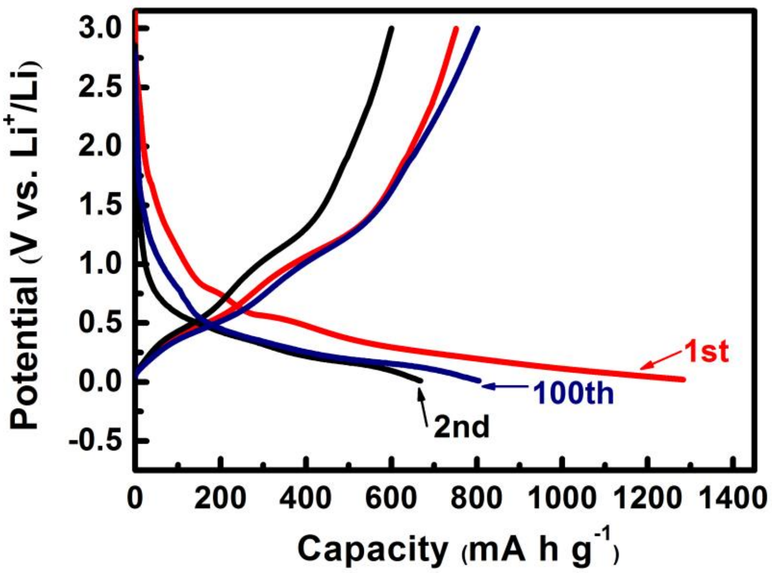

Figure S8. Galvanostatic charge–discharge profiles of the $Ca_2Ge_7O_{16}$ hollow microspheres at different cycles under a current density of 100 mA g^{-1} in electrolyte with EC/DMC/DEC (3/4/3) +5 wt% FEC.

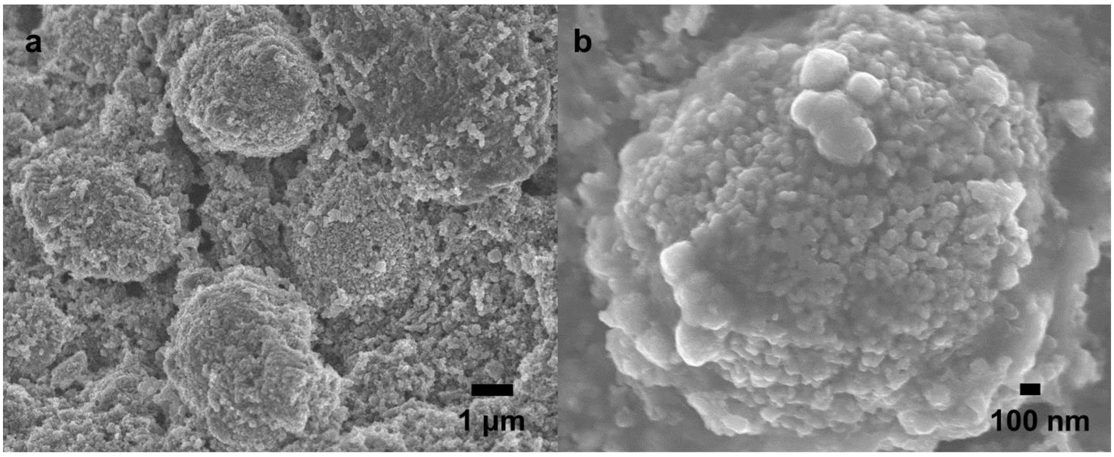

Figure S9. SEM images of $Ca_2Ge_7O_{16}$ hollow microspheres after 100 charge-discharge cycles under a current density of 100 mA g^{-1}.
